# Periorbital infections caused by Group A streptococci: a case series

**DOI:** 10.1186/s12879-025-12411-2

**Published:** 2026-01-16

**Authors:** Michaela Tinggaard, Thor Bech Johannesen, Steen Hoffmann, Aase Bengaard Andersen, Peter Bjerre Toft

**Affiliations:** 1 Department of Infectious Diseases, Copenhagen University Hospital, Rigshospitalet, Copenhagen, Denmark; 2 Department of Bioinformatics, Statens Serum Institut, Copenhagen, Denmark; 3 Bacteria, Parasites & Fungi, Infectious Disease Preparedness, Statens Serum Institut, Copenhagen, Denmark; 4 Department of Ophthalmology, Copenhagen University Hospital, Rigshospitalet, Copenhagen, Denmark; 5 Department of Clinical Medicine, Faculty of Health and Medical Sciences, University of Copenhagen, Copenhagen, Denmark

**Keywords:** Group A streptococcus, Periorbital, Pre-septal, Post-septal, Cellulitis, NSTI

## Abstract

**Background:**

A surge in Group A streptococcus (GAS) infections has been described in the post-COVID-19 pandemic period. We reviewed cases with periorbital GAS infections at our institution during a 14-month period, including cases with necrotizing soft tissue infection (NSTI).

**Methods:**

A single center retrospective case series was performed at the University Hospital of Copenhagen, Rigshospitalet, during January 2023 to February 2024 including all adult patients referred from secondary centers with a suspicion of periorbital NSTI. All cases were treated for periorbital skin and soft tissue infections with culture-confirmed group A streptococci in an eye swab, a tissue sample or blood cultures.

**Results:**

Eleven cases with a median age of 72 (range 55–84) years with periorbital GAS infection were included. Four patients had diabetes, and one patient had eyelid surgery prior to the infection. Pre-admission symptoms included pain, swelling in the periorbital area, fever and/or a sore throat. Patients presented with fever, nausea, and/or confusion and clinical exams were noticeable for erythema and edema of the periorbital area with the eyelids fully closed on the affected side. Two cases had septic shock. Based on CT scans, six cases were diagnosed with pre-septal cellulitis and five cases were suspected of post-septal NSTI, which was confirmed by surgical debridement. The GAS isolates from tissue samples or blood cultures in these five cases were of type MLST 28 / *emm* 1.0 (M1 clone). Treatment included meropenem and clindamycin (*n* = 11), intravenous immunoglobulin (*n* = 4), surgical debridement (*n* = 5) and hyperbaric oxygen therapy (*n* = 3). One patient died in the intensive care unit. Two patients have permanently reduced visual acuity.

**Conclusion:**

We report an accumulation of cases with pre- or post-septal orbital GAS infections in which the majority had no evidence of immunosuppression or a triggering event such as trauma or surgery. All cases of invasive GAS infections were caused by the M1 clone.

## Background

Infections with *Streptococcus pyogenes*, also known as Group A streptococcus (GAS), have the potential to rapidly progress and cause life-threatening complications. GAS can cause soft tissue infections such as erysipelas and cellulitis, but the infection may spread to the subcutaneous tissue and cause necrosis, referred to as necrotizing soft tissue infection (NSTI) [1]. NSTI affects the head and neck area in 2–10% of cases, which include periorbital NSTI. Reports on the incidence of periorbital NTSI are scarce but have been estimated as an annual incidence of 0.24/1.000.000 in the UK [2]. GAS has been reported as the most common causative agent in NSTI affecting the periorbital area [3, 4].

In the post-Covid-19-pandemic period from mid-2022 and onwards, surges in both non-invasive (nGAS) and invasive GAS (iGAS) infections in both children and adults have been reported in several European countries [5–10], including Denmark [11, 12]. In the beginning of 2023, we started to notice more admissions than usual at our tertiary center hospital of patients referred due to a suspicion of periorbital NSTI and in which GAS turned out to be the causative agent.

With this retrospective case series, our aim is to describe a population of 11 cases with periorbital GAS soft tissue infection and identify common features. We wish to increase awareness among clinicians on periorbital GAS infections as these may cause not only significant morbidity but also ophthalmological sequelae.

## Methods

### Study design

This is a single center retrospective case series undertaken at the University Hospital of Copenhagen, Rigshospitalet. Medical and surgical interventions were not standardized and were performed at the discretion of the treating physicians.

### Study population

Study patients were evaluated at our institution between January 1st, 2023, and February 28th, 2024. The University Hospital of Copenhagen, Rigshospitalet, is a tertiary center with services that include intensive care, infectious diseases, and ophthalmology. Denmark is divided into five regions and Rigshospitalet receives patients referred from two out of five of these regions, when specialized treatment is needed. This includes patients referred with suspected periorbital NSTI. Our case definition was patients over 18 years of age that were treated for periorbital soft tissue infections with culture-confirmed GAS at our institution. No cases were excluded.

Data on demographic information, symptoms, clinical presentation, objective eye exam, laboratory results, treatment regimens, and outcome were collected retrospectively using electronic clinical records after oral and informed consent from patients had been obtained. Follow-up data from the outpatient ophthalmological clinic included visual acuity measured with a digital Snellen chart and clinical evaluation of eyelid function. Data were retrospectively collected from time of admission until discharge from their in-hospital stay or, if attending the outpatient clinic after discharge, until June 1st, 2024. Descriptive statistics were used to summarize demographic and clinical data.

### Definitions

Microbiological identification of GAS was defined as the growth of the bacteria in a swab from the eye, a tissue sample or blood cultures. Information on the genotype of invasive GAS isolates (that is, GAS isolates cultured from tissue samples and blood cultures) was supplied from the Neisseria and Streptococcus Reference Laboratory at Statens Serum Institut, Copenhagen, DK. Isolates were sequenced on Illumina NextSeq 550 platforms, and all genotyping was performed in-silico from whole genome sequencing data. Multilocus sequence typing (MLST) was performed using ARIBA v2.14.6 based on the *S. pyogenes* MLST scheme curated by PubMLST [13]. Draft genomes were assembled with Skesa v2.2 [14], and emm types were subsequently determined using an in-house tool, which uses Blast to identify emm-alleles from the emm sequence database curated by The U.S. Centers for Disease Control and Prevention [15].

For each case, we calculated a quick Sequential Organ Failure Assessment (qSOFA), which is a score based on tachypnea, altered mental status and hypotension. It is used to identify high-risk patients for in-hospital mortality with suspected infection outside the intensive care unit [16]. Further, we calculated a laboratory risk indicator for necrotizing fasciitis (LRINEC), which is a method based on laboratory parameters for identifying early cases of NSTI [17].

Cases were classified as having had periorbital NSTI on the finding of clinically necrotic tissue during surgical debridement and histological examination of excised tissue, as retrieved from the electronic patient record. Cases were classified as having had cellulitis when NSTI was not diagnosed, and the infection had not spread beneath the subcutaneous tissue. Computer tomography (CT) scan results were used by the clinicians to evaluate whether the spread of infection was pre- or post-septal.

## Results

### Patient cohort

Eleven cases with periorbital GAS infection were included. Median age was 72 (55–84) years and the female to male ratio was 1.75:1. Table 1 shows patient characteristics, clinical presentation, microbiological diagnosis, and treatment. Three patients were previously healthy, three patients had one comorbidity, and five patients had multiple comorbidities, which included hypertension (*n* = 8), diabetes mellitus (*n* = 4), atrial flutter (*n* = 2) and granulomatosis with polyangiitis (*n* = 1).

**Table 1:** Case characteristics, treatment, and outcome

Case	1	2	3	4	5	6	7	8	9	10	11
Age interval											
50–59						x		x			
60–69			x								
70–79	x	x			x		x		x	x	
80–89				x							x
Sex	F	F	M	F	M	M	F	F	M	F	F
Symptoms before admission	Cough, sore throat, fever	Periorbital pain, fever	Redness on cheek	Sore throat	Fever and dizziness	Periorbital pain and swelling after removal of stiches above the eye	Sore throat, ear and sinus pain, nasal discharge	Periorbital swelling and pain	Periorbital pain, swelling and redness	Wound under eye, periorbital swelling	Periorbital swelling and redness
Symptom duration (days)	4	3	2	2	3	1	7	3	7	2	3
Clinical presentation on admission	Renal failure, hypotension	Nausea, confusion, fever	Fever	Nausea, fever	Fever	Fever	Nausea, vomiting, fatigue	Nausea, Headache, dizziness, aphasia	Nausea, confusion, renal failure	Vomiting, fever, confusion	Fever, renal failure
qSOFA	2	1	0	0	0	0	2	1	1	2	2
Objective eye exam	Unilateral redness, swelling, bullae. Eye closed.	Unilateral swelling. Eye closed.	Unilateral redness, swelling, necrosis. Eye closed.	Unilateral redness, swelling. Eye closed.	Unilateral redness, swelling. Eye closed.	Unilateral redness, swelling, necrosis. Eye closed.	Unilateral redness, swelling. Eye closed.	Unilateral redness, swelling, necrosis. Eye closed.	Unilateral redness, swelling. Eye closed.	Bilateral redness, swelling, bullae. Eyes closed.	Bilateral redness, swelling, necrosis. Eyes closed.
Peak CRP (mg/L)	331	277	366	164	348	238	313	446	310	280	438
LRINEC score	9	2	7	4	8	1	1	8	5	7	5
Immuno-globulins i.v.	-	+	-	-	-	+	+	+	-	-	-
Hyperbaric oxygen therapy	-	-	-	-	-	-	+	+	-	+	-
Surgery	-	-	-	-	-	-	+	+	+	+	+
Intensive care	-	-	-	-	-	-	+	-	-	-	+
Positive samples / all samples											
Swab (eye) Tissue (eyelid) Blood cultures	2/2 0/0 0/4	1/2 0/0 0/4	2/2 0/0 0/4	2/2 0/0 0/4	1/1 0/0 0/4	2/2 0/0 0/4	3/3 1/1 0/4	2/2 0/0 3/4	2/2 2/2 0/4	1/1 0/3 3/4	2/2 2/2 0/4
Genotype	NA	NA	NA	NA	NA	NA	MLST 28 / *emm* 1.0	MLST 28 / *emm* 1.0	MLST 28 / *emm* 1.0	MLST 28 / *emm* 1.0	MLST 28 / *emm* 1.0
Admission duration (days)	13	12	8	10	15	15	23	30	12	30	4 (Passed away on day 4)
Final diagnosis	Cellulitis	Cellulitis	Cellulitis	Cellulitis	Cellulitis	Cellulitis	NSTI	NSTI	NSTI	NSTI	NSTI

GAS = group A streptococci. I.v. = intravenously. MLST = multi locus sequence type. NA = not applicable. NSTI = Necrotizing soft tissue infections. qSOFA = quick sequential organ failure score

The median duration of symptoms leading up to admission was three (one to seven) days and symptoms most frequently included pain and/or swelling in the periorbital area, fever and/or a sore throat. One patient had an eyelid tumor removed seven days before symptoms were noted.

**Fig. 1:**
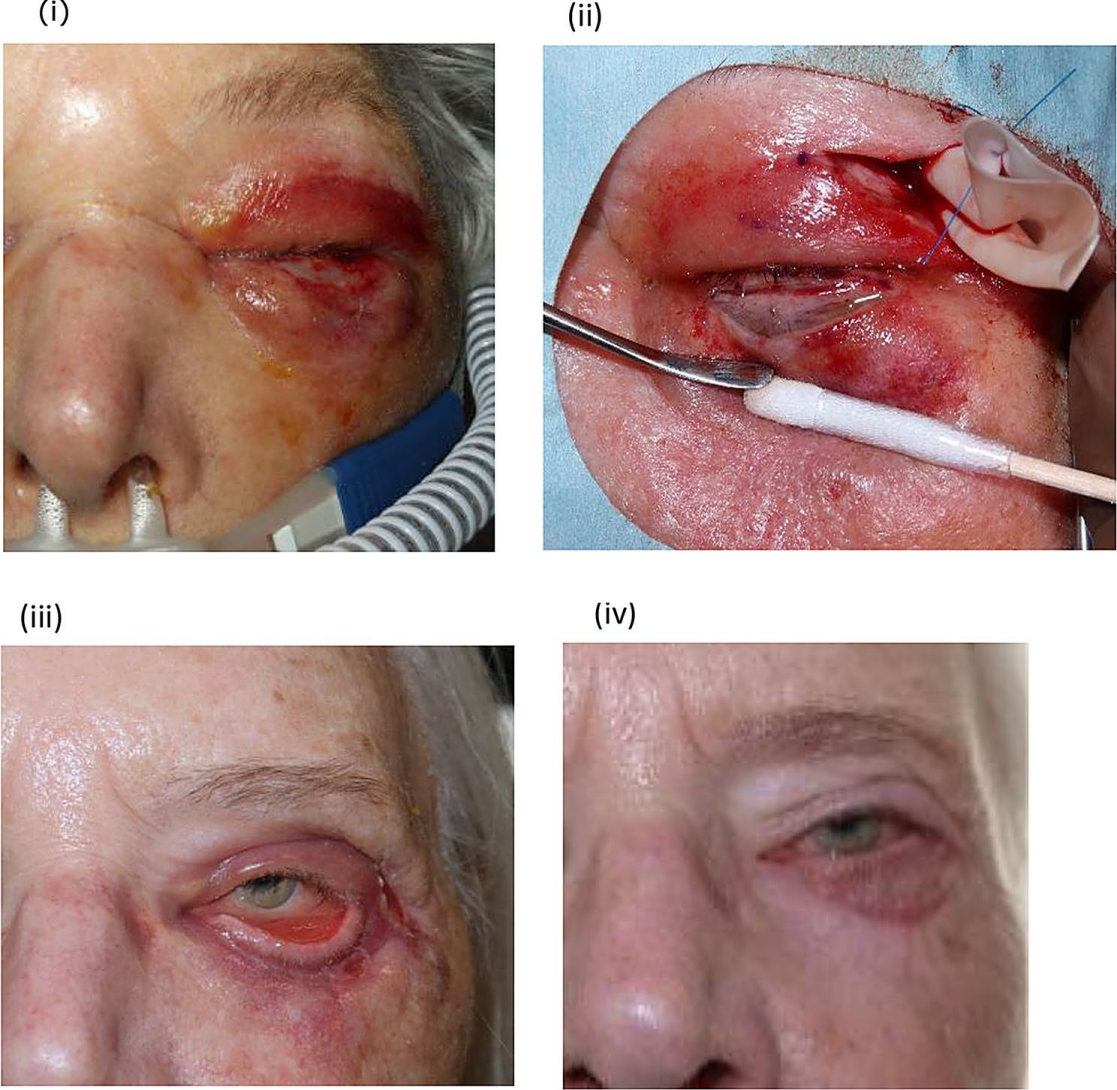
Course of a case with Group A streptococcus necrotizing soft tissue infection on (**i**) day + 1 post-admission with clinical signs of erythema and swelling of upper and lower eyelid; (**ii**) day + 1 post-admission during surgical debridement with characteristic findings of necrotizing fasciitis of the lower eyelid with murky, greyish ‘dishwater’ fluid and white tissue with thrombosed vessels (**iii**) day + 22 at discharge showing cicatricial ectropion on the lower eyelid and (**iv**) seven months post-discharge showing results from skin grafting. Pictures published with permission from case

On admission, the clinical presentation included fever (rectal temperature > 38.0 Celsius) in seven patients, nausea in five patients and confusion in three patients (Table 1). Four cases had a qSOFA score of two; three of which were cases with NSTI. Four cases had a qSOFA score of 0, all of which had a final diagnosis of cellulitis. The LRINEC scores varied from one to nine. On examination, the clinicians usually noted unilateral erythema and edema of the periorbital area with the eyelids fully closed on the affected side (Fig. 1 (i)). Two cases were noticeable for bullae on the skin and four cases for a necrotic skin lesion in the eye area as well. Three patients had acute renal failure on admission, defined as a rise in serum creatinine compared to normal levels for those patients. Finally, median peak C-reactive protein during admission was 313 (164–425) mg/L.

### Diagnosis

All patients had a CT scan shortly after admission to assess the extent of spread of the infection. In six patients, the diagnosis of pre-septal cellulitis was made based on the scan results. GAS was detected in swab samples from the affected eye(s) in these cases (Table 1). In five patients, post-septal involvement was suspected based on the CT scan and NSTI confirmed when surgical debridement was performed (Fig. 1 (iii)). In all five patients, GAS was detected in blood cultures or eyelid tissue samples. These five invasive isolates were sequenced, and all isolates were of type MLST 28 / *emm* 1.0, also referred to as the M1 clone.

### Management and outcome

The median admission time was 14 (8–30) days (Table 1). Empirical antibiotic treatment involved intravenous meropenem and clindamycin in all cases, at times preceded by one or two days of piperacillin/tazobactam or cefuroxime and metronidazole. In all cases, treatment was de-escalated to penicillin or amoxicillin as GAS was detected in the microbiological samples and antimicrobial susceptibility test results were obtained. Four cases had intravenous immunoglobulin (IVIG) in addition to antibiotics. Five cases needed surgical debridement due to NSTI and three of these cases received hyperbaric oxygen therapy (HBOT) in addition to surgery. Two of the five patients with NSTI required transfer to the intensive care unit due to septic shock with the need for vasopressor therapy.

One patient died after four days in the intensive care unit (Table 1). This patient had a do-not-resuscitate order. Ten patients attended one or more follow up visits at the outpatient ophthalmological clinic. At the latest follow up visits, two patients have permanently reduced visual acuity to 0.5 and 0.4 in the affected eye due to post-septal infection and optic neuropathy. These two patients explained that they had enjoyed normal vision before the infection. Both patients also had lower lid cicatricial ectropion (Fig. 1 (iv)) in one or both eyes, respectively. The remaining eight patients did not suffer any ophthalmological sequelae.

## Discussion

We report 11 cases of periorbital GAS infections at a tertiary hospital in Denmark during a period of 14 months, of which five were diagnosed with NSTI with GAS detected in tissue samples and/or blood cultures, classifying them as iGAS disease. Two in five regions in Denmark refer patients to the tertiary hospital and these two regions consists of approximately 2.4 million inhabitants. Compared to prior data from the UK, which has described periorbital NSTI as a very rare condition with an annual incidence of 0.24/1.000.000 [2], we consider five cases in a population of 2.4 million during 14 months as an accumulation of cases. We do not, however, have prior data on periorbital GAS infections in Denmark for comparison. Surely, our observations should be seen as a part of the surge in overall GAS cases since mid-2022. A recent report has shown that in Denmark, the monthly iGAS incidence peaked in January 2023 and was 3 times the rates seen in 2018/19, and similar trends were observed for non-invasive GAS infections [12]. Adults above 85 years of age had the highest incidence rates compared to other age groups, while children under 5 years of age experienced the highest relative increase in incidence. The cause of this post pandemic surge has been suggested to result from reduced immunity - especially in young children - as a consequence of social restrictions during the pandemic, which likely reduced the transmission of GAS [12]. Moreover, the overall increase in viral infections in the post pandemic period may have altered the susceptibility the GAS infections and thereby contributed to the surge [18].

Additionally, in Denmark, the post pandemic surge in iGAS infections has been linked to the emergence of a novel M1 sub-lineage (M1_DK_) [12]. The *emm* gene encodes the M-protein, which is a key virulence factor in GAS [19]. Type MLST 28 / *emm* 1.0 is also referred to as the M1 clone and it has been the dominant cause of invasive GAS infections in Europe in recent years [20]. The five cases of periorbital NSTI in our case series were indeed caused by the M1 clone, but we do not have data on sublineage(s).

The clinical presentation described upon admission included erythema, swelling and pain in the affected area. Further, six cases had bullae or necrotic skin lesions in the eye area on admission as well. The clinical presentation of periorbital GAS infections thus seems to be a continuum with the earliest signs being erythema and localized painful swelling of the eyelid, followed by formation of bullae and necrosis of the periorbital skin and subcutaneous tissue. However, symptom duration varied from one to three days in cases with necrosis, and necrosis may therefore be noticeable as an early manifestation of GAS infection. Two cases had visible necrosis of the skin at admission but did not have NSTI. Contrary, two cases with NSTI presented with erythema and pain, but no bullae or necrosis, despite symptoms beginning seven days prior. Erythema, swelling and pain has previously been noted as early symptoms of periorbital NSTI [21]. Thus, the clinical presentation may not be fully indicative of the spread of infection and clinicians should be alert in all cases with erythema, swelling and pain in the eye area. Finally, eight cases also presented with signs of systemic infection (nausea, confusion, renal failure), which sometimes precede skin lesions in NSTI [21].

Our data suggest that there was no triggering event prior to all but two cases of periorbital GAS infections. Contrary, in a retrospective multicenter case series of periorbital NSTI from the UK and Australia, 75% of 29 cases recalled minor periocular trauma or an infected lesion prior to the spread of infection [4]. Similarly, 50–75% of cases of periorbital NSTI in other prior case series or reviews were preceded by an event such as trauma, surgery, or an infected lesion [2, 3, 22, 23]. The reason for the discrepancy compared to our case series is not clear. It could be that the patients included here simply did not recall a precipitating event, did not mention it to the treating physician or that it was not stated in their electronic record. It could also be speculated that novel lineages of GAS are more invasive and, to an even higher degree than prior lineages, do not require a clear skin lesion to spread. Three cases had a sore throat prior to admission, which could indicate that the origin of infection was pharyngitis caused by GAS.

Four cases had diabetes, which in previous studies has been found to be a risk factor for invasive GAS disease [24] and periorbital NSTI [21]. When focusing on the five patients diagnosed with NSTI, two of these had no comorbidities, one was treated for hypertension, and the two remaining cases both had diabetes. Hence, in the majority of cases, there was no evidence of immunosuppression. Other case reports on periorbital NSTI have found more than half of cases to have one or more immunosuppressing condition(s) such as cancer, chronic alcohol disease, diabetes or rheumatic disease [3, 4, 23]. A possible explanation is that all cases included in our case series were caused by a single organism, which is referred to as type II NSTI, whereas type I NSTI is polymicrobial. It has been stated that type I NSTI is more often associated with immunosuppression than type II NSTI [3], although the evidence for this statement seems unclear.

No obvious trend was observed for the LRINEC score in cases overall nor cases with and without NSTI (Table 1). Hence, our case series do not support the use of this score to rule in or rule out NSTI in periorbital GAS infections. In regards to the qSOFA score, three in six cases with cellulitis had a score of 0, while a score of 0 was not seen among NSTI cases. In other words, all NSTI cases showed signs of systemic infection with either tachypnea, hypotension or altered mental status. However, two in six cases with pre-septal cellulitis had a qSOFA score of 1–2, indicating a greater risk for a poor outcome, although this was not the case. Accordingly, neither qSOFA or LRINEC seemed to be highly sensitive for assessing risk of mortality or NSTI. However, no clear conclusions can be drawn due the relatively small number of cases in this series.

This case series illustrates the risk of severe morbidity in periorbital GAS infections, including sepsis with the need for intensive care treatment in 18% of cases, the need for surgical debridement in 45% of cases, ophthalmological sequelae in 18% of cases and mortality in 9% of cases. Thus, the management of periorbital GAS infections often requires transfer to a center with the possibility of involving ophthalmologists, infectious disease/microbiology specialists and intensive care specialists for optimal care. It is important that clinicians are aware of signs of periorbital infection to rapidly initiate treatment with appropriate antibiotics, which includes β-lactam antibiotics with the addition of clindamycin. Clindamycin is a protein synthesis inhibitor and has the ability to down-regulate bacterial production of toxins and has been shown to improve survival when added to β-lactam antibiotics in invasive GAS infections [25]. A recent study has shown linezolid to be non-inferior to clindamycin as an adjunctive to β-lactam antibiotics in iGAS infections - suggesting linezolid as an alternative anti-toxin therapy in countries with concerns of clindamycin resistance [26, 27], but these results are based on retrospective data.

HBOT and IVIG were received in some cases. HBOT might increase tissue oxygen tension in infected necrotic wounds and potentiate antibiotic efficacy, while IVIG may enhance bacterial opsonization and toxin neutralization [21]. However, reviews conclude that there is insufficient data to support the routine use of these therapies in NSTI [21, 28].

This case series provides useful information on the clinical characteristics and findings of patients with periorbital GAS infections in the post-pandemic period. Questions remain as to whether other countries have experienced a similar rise in periorbital GAS infections, whether the clinical characteristics differ from past periorbital GAS infections and whether we will continue to notice more cases than in the pre-pandemic years.

## Conclusion

Our findings indicate an accumulation of cases at our institution with pre- or post-septal orbital GAS infections caused by the M1 clone in individuals without identifiable risk factors or triggering events. These findings highlight the need for continued epidemiological surveillance in the coming years.

## Data Availability

All relevant data is included in the manuscript. A separate data repository is not applicable.

## Abbreviations

CT: Computer tomography
GAS: Group A Streptococcus
HBOT: Hyperbaric oxygen therapy
iGAS: Invasive group A streptococcus
IVIG: Intravenous immunoglobulin
LRINEC: Laboratory risk indicator for necrotizing fasciitis
nGAS: Non-invasive group A streptococcus
NSTI: Necrotizing soft tissue infection
qSOFA: Quick Sequential Organ Failure Assessment
